# cGMP/cGKI signaling in peripheral vascular smooth muscle does not involve TrpC3 or TrpC6 channels

**DOI:** 10.1186/1471-2210-11-S1-O25

**Published:** 2011-08-01

**Authors:** Jörg Wegener, Florian Loga, Katrin Domes, Franz Hofmann

**Affiliations:** 1 Institut für Pharmakologie und Toxikologie, TU München, Germany

## Background

Signaling by intracellular cGMP and cGMP-dependent protein kinase I (cGKI) is the major pathway in vascular smooth muscle, by which endothelial NO regulates vascular tone. The most important targets of cGKI include the myosin-interacting subunit of myosin phosphatase 1, the regulator of G-protein signaling 2, the inositol receptor associated cGKI-substrate, and the BK channel. Recent evidence suggest that TrpC channels are also targets of cGKI in smooth muscle and mediate, at least partially, the relaxant effects of cGMP.

## Results

We tested this new concept by investigating the role of cGMP/cGKI signaling on vascular tone and peripheral resistance using cGKI-, TrpC6-, and TrpC3-knock-out mice. We found no differences in the response to alpha-adrenergic stimulation with respect to the contractility of thoracic aorta or to the increase in peripheral resistance using preparations from the knock-out models. Activation of cGKI by 8-Br-cGMP diminished aortic tone and peripheral resistance to a similar extent in control, TrpC6-/-, and TrpC3-/- mice. No effect of 8-Br-cGMP was observed in preparations from smooth-muscle specific cGKI-/- mice.

## Conclusion

The results suggest that cGMP/cGKI signaling in aorta and peripheral vessels from mice does not require TrpC6 or TrpC3 channels.

